# Development and validation of a contrast-enhanced CT-based radiomics nomogram for preoperative diagnosis in neuroendocrine carcinoma of digestive system

**DOI:** 10.3389/fendo.2023.1155307

**Published:** 2023-04-12

**Authors:** Liang Xu, Xinyi Yang, Wenxuan Xiang, Pengbo Hu, Xiuyuan Zhang, Zhou Li, Yiming Li, Yongqing Liu, Yuhong Dai, Yan Luo, Hong Qiu

**Affiliations:** ^1^ Department of Oncology, Tongji Hospital, Tongji Medical College, Huazhong University of Science and Technology, Wuhan, China; ^2^ Department of Otolaryngology-Head and Neck Surgery, Tongji Hospital, Tongji Medical College, Huazhong University of Science and Technology, Wuhan, China; ^3^ Department of Radiology, Tongji Hospital, Tongji Medical College, Huazhong University of Science and Technology, Wuhan, China

**Keywords:** radiomics, contrast-enhanced CT, neuroendocrine carcinoma, diagnosis model, digestive system

## Abstract

**Objectives:**

To develop and validate a contrast-enhanced CT-based radiomics nomogram for the diagnosis of neuroendocrine carcinoma of the digestive system.

**Methods:**

The clinical data and contrast-enhanced CT images of 60 patients with pathologically confirmed neuroendocrine carcinoma of the digestive system and 60 patients with non-neuroendocrine carcinoma of the digestive system were retrospectively collected from August 2015 to December 2021 at Tongji Hospital, Tongji Medical College, Huazhong University of Science and Technology, and randomly divided into a training cohort (n=84) and a validation cohort (n=36). Clinical characteristics were analyzed by logistic regression and a clinical diagnosis model was developed. Radiomics signature were established by extracting radiomic features from contrast-enhanced CT images. Based on the radiomic signature and clinical characteristics, radiomic nomogram was developed. ROC curves and Delong’s test were used to evaluate the diagnostic efficacy of the three models, calibration curves and application decision curves were used to analyze the accuracy and clinical application value of nomogram.

**Results:**

Logistic regression results showed that TNM stage (stage IV) (OR 6.8, 95% CI 1.320-43.164, *p*=0. 028) was an independent factor affecting the diagnosis for NECs of the digestive system, and a clinical model was constructed based on TNM stage (stage IV). The AUCs of the clinical model, radiomics signature, and radiomics nomogram for the diagnosis of NECs of the digestive system in the training, validation cohorts and pooled patients were 0.643, 0.893, 0.913; 0.722, 0.867, 0.932 and 0.667, 0.887, 0.917 respectively. The AUCs of radiomics signature and radiomics nomogram were higher than clinical model, with statistically significant difference (Z=4.46, 6.85, both *p* < 0.001); the AUC difference between radiomics signature and radiomics nomogram was not statistically significant (Z=1.63, *p* = 0.104). The results of the calibration curve showed favorable agreement between the predicted values of the nomogram and the pathological results, and the decision curve analysis indicated that the nomogram had favorable application in clinical practice.

**Conclusions:**

The nomogram constructed based on contrast-enhanced CT radiomics and clinical characteristics was able to effectively diagnose neuroendocrine carcinoma of the digestive system.

## Introduction

Neuroendocrine neoplasms (NENs) are rare tumors arising from neuroendocrine cells and peptidergic neurons, which are characterized by secreting biogenic amines and various peptide hormones (1). They can develop in almost any organ of the body, mainly in the digestive and respiratory systems, such as the esophagus, gastroenteropancreas and lung tissues, and the biology of the disease is highly heterogeneous (2). Although relatively rare, the incidence of NENs has been increasing, with a more than 6-fold increase over a 40-year period, particularly in the digestive system (3). The latest 2019 WHO guidelines classified NENs into poorly differentiated and highly aggressive neuroendocrine cancers (NECs) and highly differentiated and inert neuroendocrine tumors (NETs) based on mitotic rate and Ki-67 index (4).

Due to the unspecific clinical symptoms of NECs of digestive system, it is prone to misdiagnose NECs as adenocarcinomas or squamous carcinomas before surgery in clinical practice. The low-differentiated digestive system NECs are highly malignant and aggressive, and most patients have distant metastasis at the time of diagnosis (5). For patients with combined distant metastasis, surgery does not benefit due to the rapidly progressive biology of NECs, and platinum-based chemotherapy is the primary recommended first-line treatment option. In the case of locally advanced non-NECs such as adenocarcinomas or squamous carcinomas of the digestive system, surgery is still an important treatment modality. In addition, the prognosis of NECs is also significantly worse compared to non-NECs. If the tumor can be diagnosed preoperatively, it will be beneficial to select a more suitable treatment modality and judge the prognosis. Currently, NECs in the digestive system are clearly diagnosed by postoperative pathological findings, and there is still a lack of effective and definitive methods for preoperative diagnosis. Therefore, exploring an effective new method for preoperative diagnosis is crucial for clinical practice.

Contrast-enhanced CT is one of the most common and important imaging examinations for diagnosing tumor of the digestive system. Medical images contain a large amount of invisible data, and it is the value of radiomics to reveal these invisible disease features. Radiomics has been defined as the use of mathematical algorithms to transform the underlying pathophysiological information contained in medical images into quantitative, high-dimensional image features and to explore the correlation of these image features with clinical outcomes or biological properties (6, 7). When radiomics is applied to cancer research, it is possible to characterize the imaging of tumor patients non-invasively, quantify the heterogeneity between tissues, describe the microenvironment of the tumor, assess the effectiveness of treatment, and predict survival after obtaining radiological images by CT, MRI, and other examination methods (8, 9).

In recent years, radiomics has been gradually and widely used in the diagnosis of cancers (10), identification of molecular typing of tumors (11), prediction of survival status of patients (12), and the use of imaging genomics to analyze the relationship between imaging features and genomic features to dissect tumor heterogeneity (13). Radiomics studies targeting NETs are also increasing, and radiomics can be applied in the diagnosis of pancreatic NETs (14), predicting the grading of pancreatic NETs (15), determining the prognosis of NETs (16), and assessing the effects of drug therapy for NETs (17). However, there are few radiomics studies for NECs, *Wang* et al. (18) identified gastric NECs from gastric adenocarcinoma with CT radiomics. To our knowledge, there are no radiomics studies for other digestive system NECs such as esophageal, intestinal and pancreatic. Therefore, we aim to conduct a study to extract tumor radiomics features based on contrast-enhanced CT images and construct a nomogram in combination with clinicopathological characteristics to diagnose NECs of the digestive system before surgery.

## Materials and methods

### Patients

This retrospective study was approved by the Medical Ethics Review Committee of Tongji Hospital, Tongji Medical College, Huazhong University of Science and Technology, and written consent was waived. The inclusion criteria were as follows: patients with pathological diagnosis of esophageal or gastroenteropancreatic NEC by surgery or biopsy; CT examination within 2 weeks before surgery or biopsy. The exclusion criteria were as follows: receiving the corresponding treatment before the contrast-enhanced CT examination; No contrast-enhanced CT examination or unavailability of contrast-enhanced CT image data; poor image quality affecting image segmentation and evaluation.

A total of 177 patients pathologically-diagnosed NECs of the digestive system from August 2015 to December 2021 were identified from the hospital database. According to the above inclusion and exclusion criteria, 60 patients with NEC of the digestive system were finally included, including 23 esophageal NECs, 22 gastric NECs, 6 intestinal NECs, and 9 pancreatic NECs. The same number of adenocarcinomas or squamous carcinomas of the digestive system at the same sites were systematically sampled and matched as a control group for NECs. Patients were randomized in a 7:3 ratio into a training cohort (n=84) and a validation cohort (n=36) (**Supplementary Figure 1**).

### Image acquisition

All 120 patients underwent contrast-enhanced CT examination within 2 weeks before surgery or biopsy using a 64-slice MDCT system (Discovery C750 HD, GE Healthcare). Patients were trained to breathe and hold their breath before the scanning examination. The patient was advised to be in a supine position during the examination, and the patient was told to over-supine the neck and lower the shoulders as much as possible during the scan and avoid swallowing movements.

Contrast-enhanced CT scans were performed by injecting non-ionic iodinated contrast agent Iopromide (Ultravist, Bayer Healthcare, Wayne, NJ, iodine concentration of 370 mg/mL) at a flow rate of 3.0-3.5 mL/s *via* the anterior elbow vein. Contrast-enhanced chest CT was acquired 15 seconds after injection. Bolus tracking technique was used for contrast-enhanced abdominal CT and arterial phase was automatically triggered 5-8 seconds after the attenuation of abdominal aorta reached 150 HU. The main scanning parameters were as follows: tube voltage 100-120 kV, rotation time 0.5- 0.6 s, tube current 200-350 mA, and slice thickness 5 mm. The acquired raw data were reconstructed to a slice thickness of 1.25 mm and exported in DICOM format for analysis.

### Image segmentation and radiomics feature extraction

On the picture archiving and communication system, two experienced radiologists reviewed the contrast-enhanced CT images and discussed together to determine the tumor location with reference to endoscopy and other findings. Arterial phase images of the contrast-enhanced CT were used for image segmentation and radiomics feature extraction. Segmentation was performed by two experienced oncologist and radiologist who were blind to clinical information according to the tumor location recorded by the two radiologists. The 3D Slicer image computing platform (version 5.0.3) software was used to manually segment the 3D volume of interest (VOI) of the entire tumor, and the cystic or necrotic areas were avoided during the segmentation.

A total of 107 features, including First order features, Shape features (3D), Shape features (2D), Gray level co-occurrence matrix (GLCM) features, Gray level size zone matrix (GLSZM) features, Gray level run length matrix (GLRLM) features, Neighbouring gray tone difference matrix (NGTDM) features and Gray level dependence matrix (GLDM) features were extracted using the “Slicer Radiomics” extension package of 3D Slicer software. To determine the intra- and inter-reader reproducibility of radiomics features, 20 randomly-selected cases were segmented by the oncologist after a period of 1 month and by radiologist with 5 years of experience.

### Radiomics feature selection and radiomics signature development

Radiomics features extracted from the images were subjected to Z-score normalization. Intraclass correlation coefficients (ICC) were calculated and features with ICC > 0.75 in intra- and inter-reader reproducibility tests were considered reproducible and include in feature selection. In the R software (version 4.2.0, http://www.r-project.org), the least absolute shrinkage and selection operator (LASSO) logistic regression algorithm using the “glmnet” package was used to select features that were closely associated with the diagnosis of NECs of the digestive system. The features in the training cohort that were strongly correlated with the diagnosis of NECs of the digestive system were screened by a 10-fold cross-validation.

Based on the linear combination of the screened features and their correlation coefficients, radiomics score (Rad-score) was calculated. Receiver operating characteristic (ROC) curves were plotted to analyze the efficacy of radiomics signature for diagnosing NEC of the digestive system.

### Clinical model and clinical-radiomics model development

Clinical characteristics including age, gender, TNM stage, preoperative CEA and preoperative CA199 were compared between NECs and non-NECs of the digestive system, and factors with statistical significance were further included into multivariable logistic regression analysis to establish a clinical model.

The clinical features associated with the diagnosis of NECs were combined with radiomics signature using multivariable logistic regression analysis to build a clinical-radiomics model, and a nomogram based on these clinical-radiomics model was also built. ROC curves were plotted to assess the discrimination of the models, and Delong’s test was used to compare the area under the curve of different models. Calibration curves were used to estimate the accuracy of the nomogram, and decision curve analysis (DCA) was used to assess the clinical utility of the nomogram.

### Statistical analysis

All statistical analyses were performed in R software. The χ² test was used for the comparison of categorical data, and the t-test was used for the comparison of quantitative data. The “Glm” package of the R software package was used for logistic regression analysis, the “Glmnet” package was used for LASSO regression algorithm analysis, and the “pROC” package was used for ROC curves plotting. The calibration curve and DCA were executed using the “Rms” and “rmda” packages, respectively. The differences were statistically significant at *p*<0.05.

## Results

### Patient characteristics and clinical model construction and validation

There were 84 patients in the training cohort, among which 42 were NECs and 42 were non-NECs; there were 36 patients in the validation cohort, among which 18 were NECs and 18 were non-NECs. In the training and validation cohorts, the differences in TNM stage between the NEC and non-NEC groups were statistically significant (*p*<0.05), while the differences in clinical characteristics such as age, gender, preoperative CEA and CA199 were not statistically significant (all *p*>0.05), as shown in **Table 1**. In the training cohort, logistic regression was performed on TNM stage, and the results showed that only TNM stage (stage IV) (OR 6.8, 95%CI 1.320-43.164, *p*=0.028) was an independent factor for the diagnosis of NECs, and the variables and coefficients of the clinical model are shown in **Supplementary Table 1**. The clinical model was constructed from TNM stage (stage IV).

**Table 1:** Patient clinical characteristics in the training and validation cohorts.

Characteristics	Training cohort			Validation cohort		
	NEC	Non-NEC	*p*-value	NEC	Non-NEC	*p*-value
Age(year), mean ± SD	64.02 ± 9.02	56.24 ± 9.69	0.606	62.72 ± 11.21	56.83 ± 9.488	0.762
Sex			0.503			1.000
Female	27	24		3	3	
Male	15	18		15	15	
CEA^*^(ng/ml)			0.357			1.000
<5	27	35		10	12	
≥5	8	6		4	4	
CA199^*^(u/ml)			0.281			0.426
<37	29	32		12	10	
≥37	2	6		1	4	
TNM			0.012			0.014
I	3	6		1	1	
II	8	17		6	10	
III	14	14		2	6	
IV	17	5		9	1	

*represents the presence of missing values.

**Figure 1:**
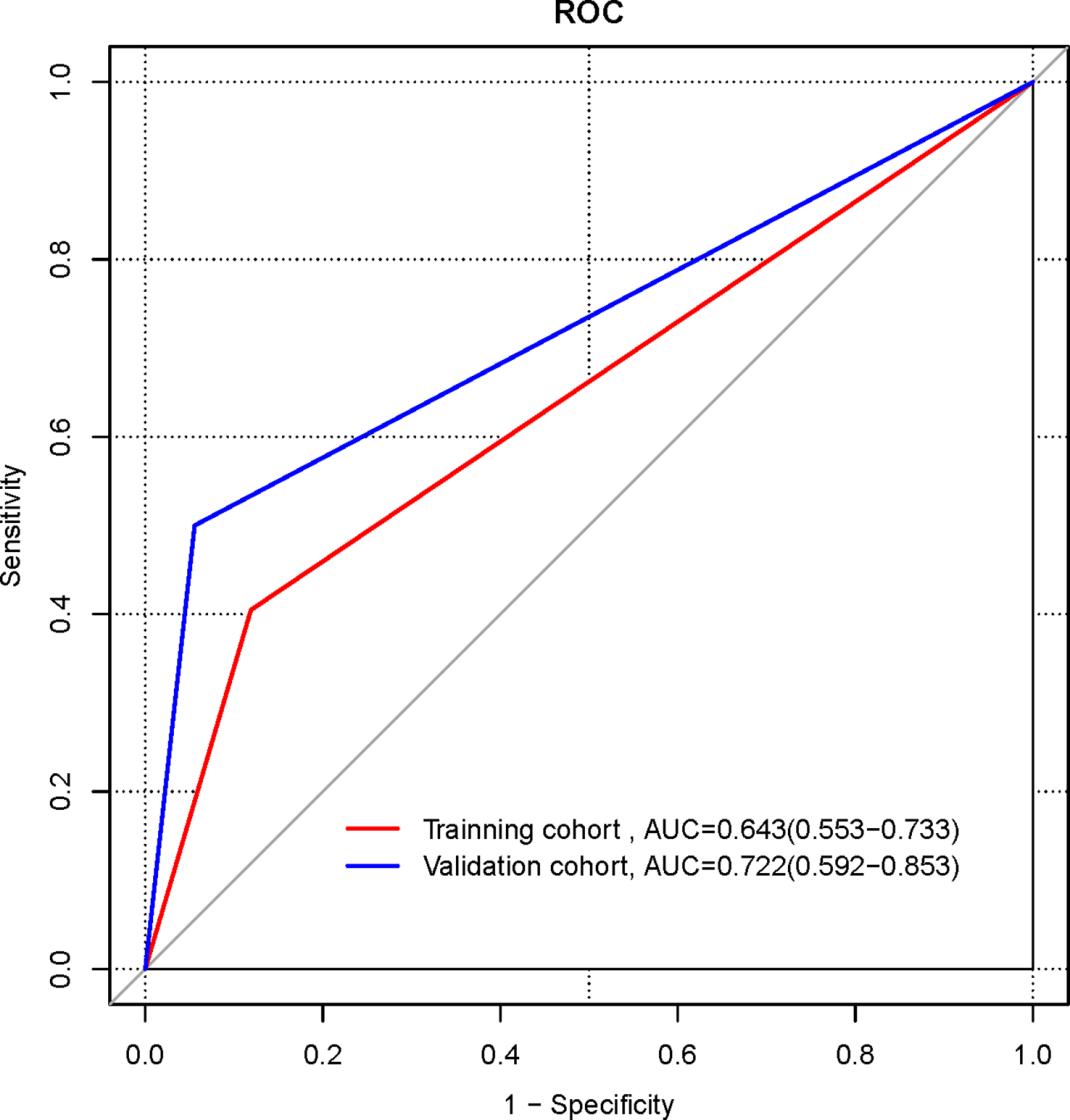
ROC curves of clinical model in the training and validation cohorts.

The ROC curves of the clinical models in the training and validation cohorts were plotted (**Figure 1**). In the training cohort, the AUC of the clinical model for diagnosing NECs is 0.643 (95%CI 0.553-0.733), the sensitivity is 0.405, the specificity is 0.881. In the validation cohort, the AUC of the clinical model for diagnosing NECs is 0.722 (95%CI 0.592-0.853), the sensitivity is 0.500, the specificity is 0.944.

### Radiomics signature construction and validation

**Figure 2:**
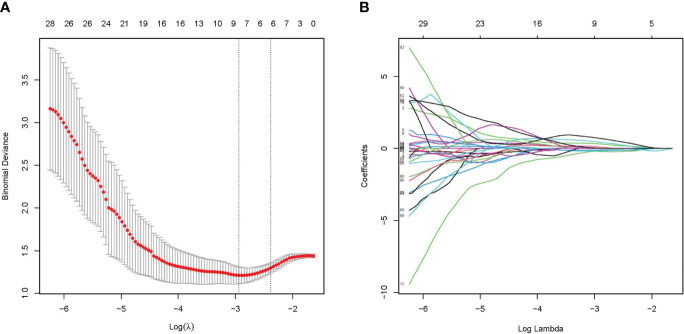
Radiomics feature selected by LASSO regression algorithm. **(A)** Plotting of multinomial deviance versus log(λ). **(B)** LASSO coefficient profiles of the radiomics features.

A total of 107 radiomics features were extracted, and the consistency assessment showed that the ICC of all radiomics features was >0.75. The best radiomics features with six non-zero coefficients in the training cohort were determined by the LASSO regression algorithm (**Figure 2**) to be closely related to the diagnosis of NECs, and the best value of the LASSO adjustable parameter (λ) was 0.092. These six radiomics features and their corresponding coefficients were linearly combined to construct the radiomics signature with the following equation: Rad-score= 0.00885470+ (0.15453837 × LeastAxisLength) – (0.18987915 × SurfaceVolumeRatio) – (0.10557837 × Uniformity) + (0.15860176 × InverseVariance) + (0.35593795 ×MCC) + (0.11645836 × LargeDependenceLowGrayLevelEmphasis).

**Figure 3:**
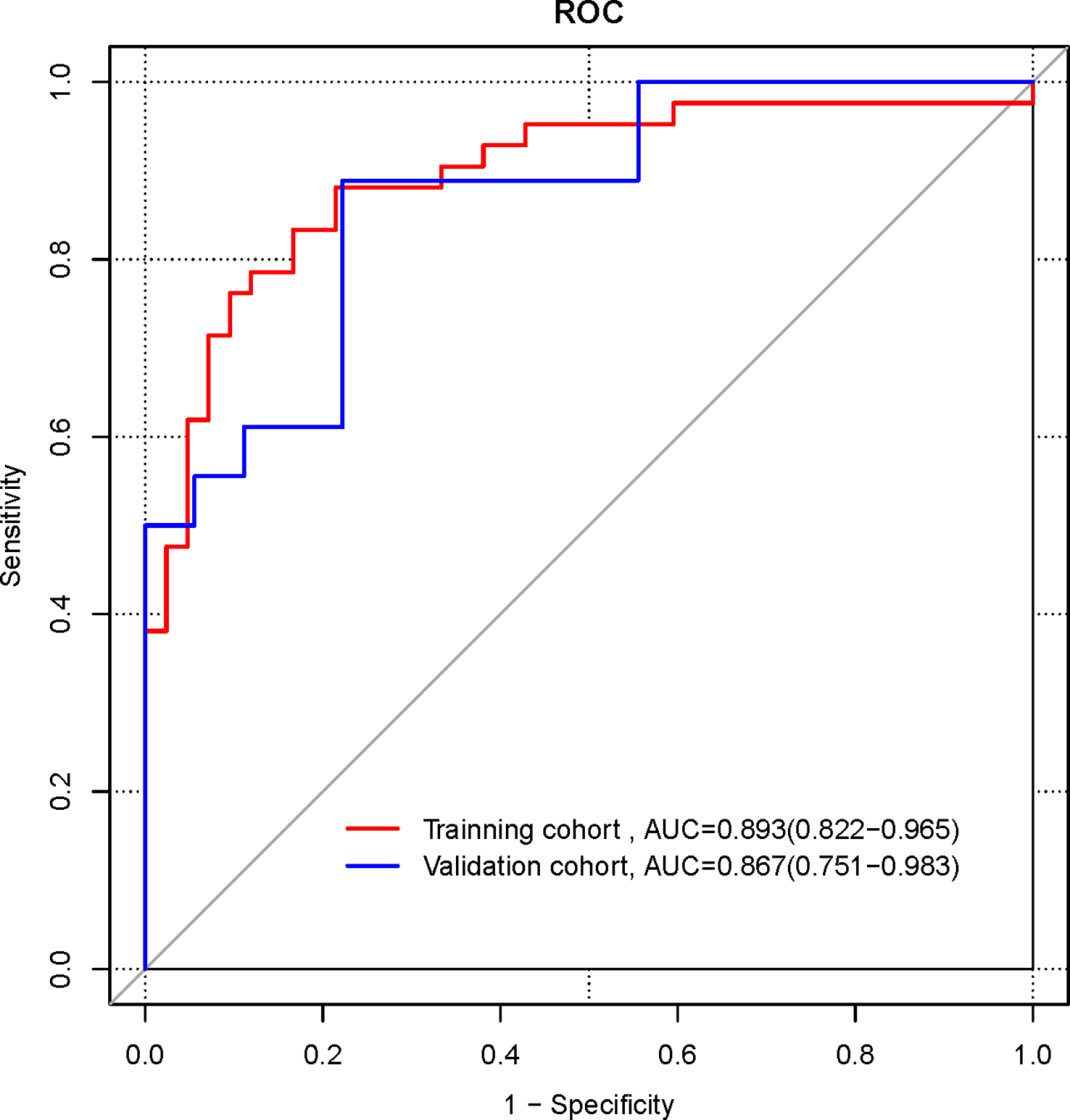
ROC curves of radiomics signature in the training and validation cohorts.

The ROC curves were plotted for the Radiomics signature (**Figure 3**). In the training cohort, the AUC of the radiomics signature for diagnosing NECs is 0.893 (95%CI 0.822-0.965), the sensitivity is 0.833, the specificity is 0.833. In the validation cohort, the AUC of the radiomics signature for diagnosing NECs is 0.867 (95%CI 0.751-0.983), the sensitivity is 0.889, the specificity is 0.778.

### Nomogram construction and validation

**Figure 4:**
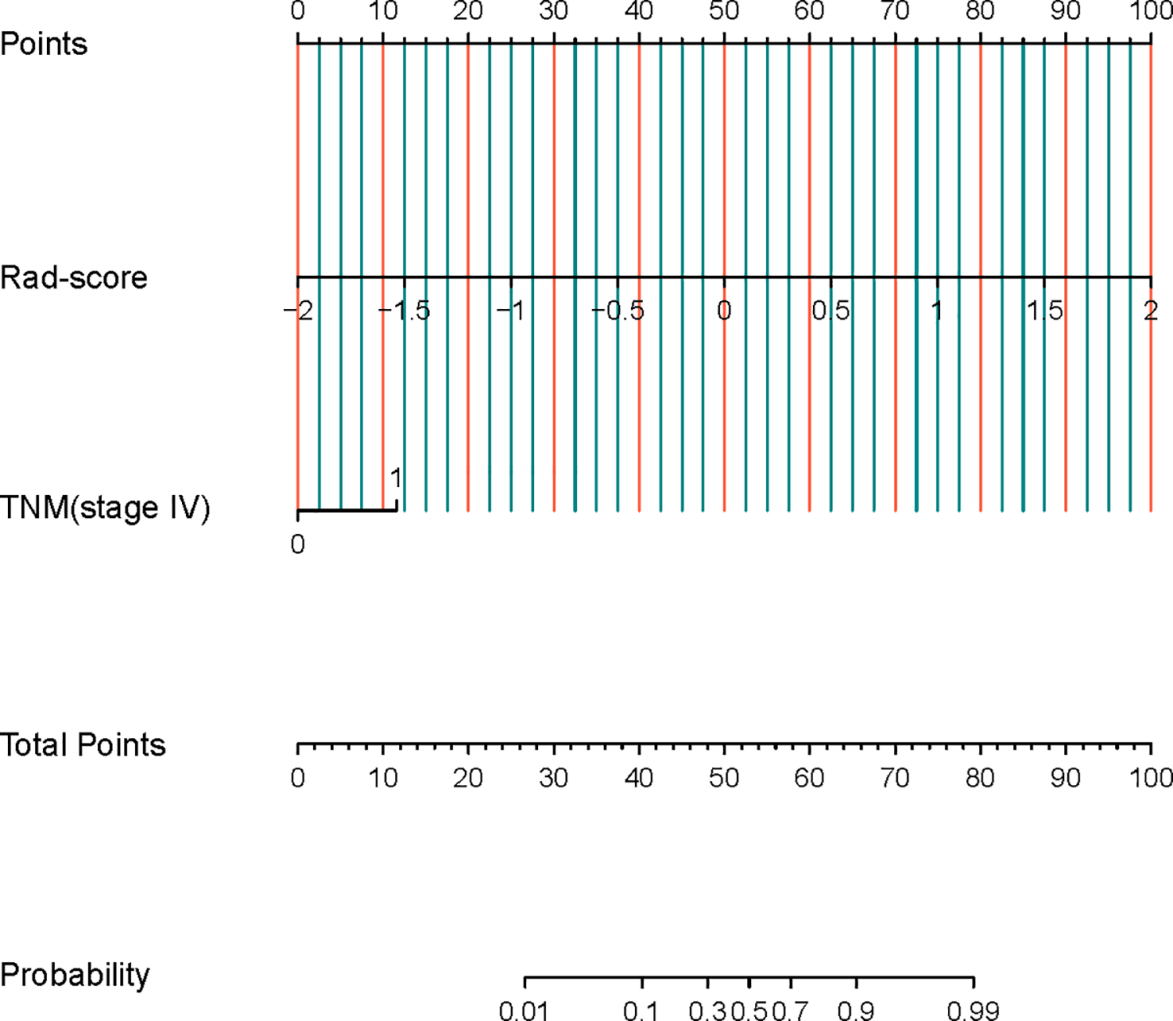
Radiomics nomogram constructed based on clinical model and radiomics signature.

Logistic regression analysis showed that both radiomics signature (OR 56.869, 95% CI 11.354-471.239, p<0.001) and TNM stage (stage IV) (OR 5.03, 95% CI 1.741-16.937, *p*=0.005) were independent predictors for the diagnosis of NECs of the digestive system, and a combined clinical-radiomics diagnostic model containing these two predictors was constructed to generate a radiomics nomogram(**Figure 4**).

**Figure 5:**
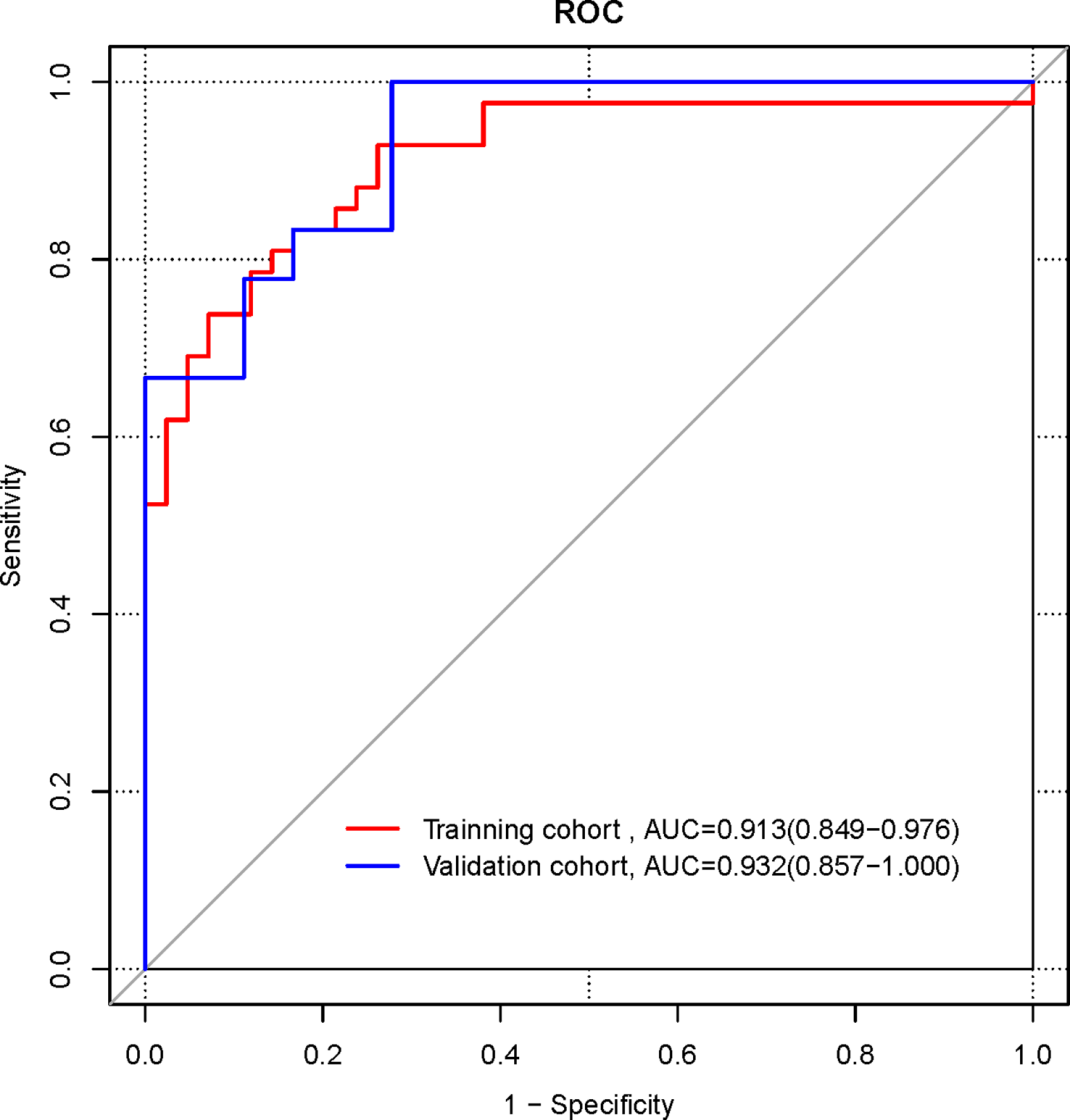
ROC curves of radiomics nomogram in the training and validation cohorts.

The ROC curves were plotted for the nomogram (**Figure 5**). In the training cohort, the AUC of the radiomics nomogram for diagnosing NECs is 0.913 (95%CI 0.849-0.976), the sensitivity is 0.833, the specificity is 0.833. In the validation cohort, the AUC of the radiomics nomogram for diagnosing NECs is 0.932 (95%CI 0.857-1.000), the sensitivity is 1.000, the specificity is 0.722.

**Figure 6:**
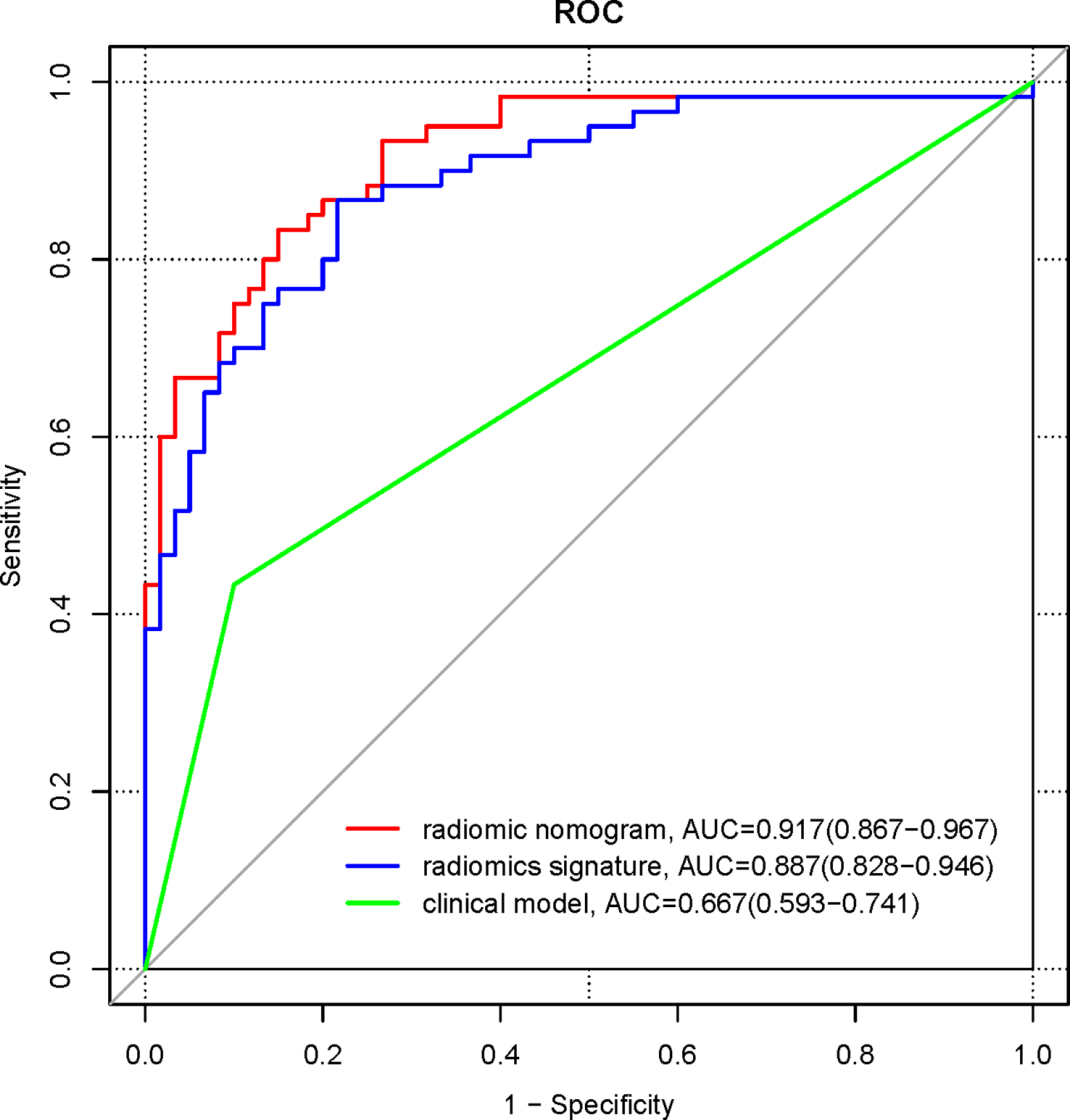
ROC curves for clinical model, radiomics signature, and radiomics nomogram in the pooled population.

The ROC curves were plotted for the clinical model, radiomics signature, and radiomics nomogram in the pooled population (**Figure 6**). The AUC of the clinical model for diagnosing NECs is 0.667 (95%CI 0.593-0.741), the sensitivity is 0.433, the specificity is 0.9. the AUC of the radiomics signature for diagnosing NECs is 0.887 (95% CI 0.828-0.946), the sensitivity is 0.867, the specificity is 0.783. the AUC of the radiomics nomogram for diagnosing NECs is 0.917 (95%CI 0.867-0.967), the sensitivity is 0.833, the specificity is 0.85. The diagnosis performance of three models in the training and validation cohort is shown in **Supplementary Table 2**.

Delong’s test was used to compare the significance of the AUCs of the three different models. The results showed that the AUC of the radiomics signature and the radiomics nomogram were higher than those of the clinical model, and the differences were statistically significant (Z=4.46, 6.85, both *p*<0.001); the difference in the AUC of the radiomics signature and the radiomics nomogram were not statistically significant (Z=1.63, *p* =0.104).

**Figure 7:**
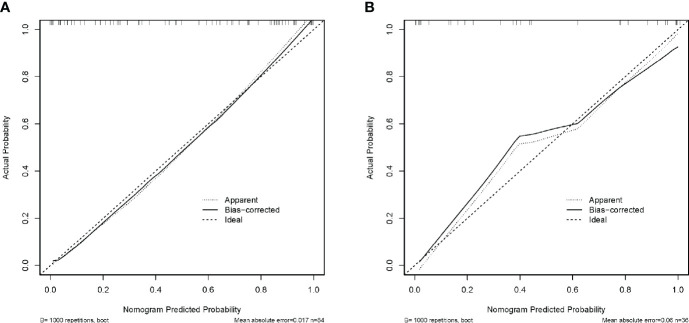
Calibration curves of the nomogram in the training **(A)** and validation **(B)** cohorts.

Calibration curves were developed to verify the discriminative efficacy of the nomogram, and the mean absolute error of the calibration curves for the training cohort was 0.017 (**Figure 7A**); the mean absolute error of the calibration curves for the validation cohort was 0.06 (**Figure 7B**). The calibration curve was close to the ideal curve, which indicated that the prediction of the constructed nomogram for the diagnosis of NECs of the digestive system fitted well with the actual results.

**Figure 8:**
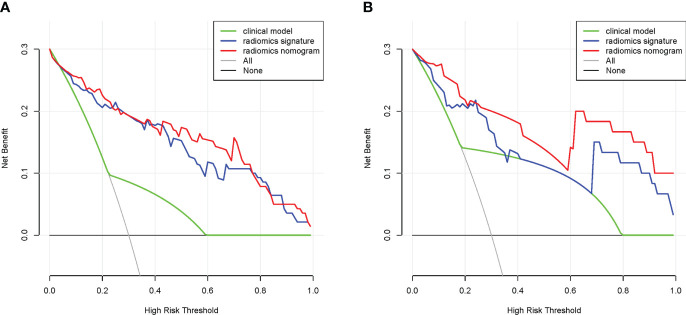
DCA of the nomogram in the training **(A)** and validation **(B)** cohorts.

DCA was used to verify the value of the nomogram for clinical applications, and the results showed that in the training cohort, DCA in the 4%-99% threshold range was more effective in diagnosing NECs using radiomics signature or radiomics nomogram than using clinical model (**Figure 8A**). In the validation cohort, DCA in the 1%-35%, 37%-39%, and 69%-99% threshold range was more effective in diagnosing NECs using radiomics signature than using clinical model, DCA in the 1%-99% threshold range was more effective in diagnosing NECs using radiomics nomogram than using clinical model (**Figure 8B**). This suggested that radiomics signature or radiomics nomogram had great clinical application.

## Discussion

In this study, we developed and validated a new radiomics nomogram for preoperative diagnosis of NECs and non-NECs in the digestive system. The radiomics nomogram, which combines radiomic signature and TNM stage (stage IV), could be an effective method diagnosing NECs of the digestive system.

Due to the significant differences in treatment and prognosis between NECs and non-NECs, preoperative diagnosis is significant for the treatment options and prognosis of patients. At present, there is still a lack of effective methods. CT examination is an important examination for the diagnosis of cancer, which can not only detect cancer lesions but is also essential for the clinical staging of cancer (19), and contrast-enhanced CT will be more obvious. Contrast-enhanced CT is largely able to reflect the status of microcirculation inside the cancer, which could understand the differences between different cancers and judge the nature of the cancer (20, 21). The internal blood supply is overly adequate in most NECs (22), which means that it is possible to detect differences between NECs and non-NECs by contrast-enhanced CT, and contrast-enhanced CT is potentially an effective tool for diagnosing NECs.

Radiomics has a good application in the diagnosis of cancer by extracting information from the inner data of CT and MRI images, and the image features reflect the underlying pathophysiological changes to a certain extent, which can reflect the internal heterogeneity of cancers noninvasively and at low cost (23, 24). Radiomics analysis has also shown good clinical value in NETs.

Clinical characteristics of age, gender, TNM stage, preoperative CEA and preoperative CA199 were included in this study to explore the role of clinical characteristics in the diagnosis for NECs of the digestive system. The results showed that TNM stage (stage IV) was an independent predictor for the diagnosis of NECs. Stage IV indicated a higher possibility of diagnosis of NECs. This is consistent with the biological characteristics of NECs, which is highly malignant, with the majority having developed distant metastases at the time of diagnosis (5), and has mostly developed into stage IV at the time of diagnosis. However, we constructed clinical models with relatively low AUCs developed from TNM stage (stage IV) in the training cohort, validation cohort, and pooled population, 0.643, 0.722, and 0.691, respectively, suggesting the relatively limited predictive value of clinical model.

In this study, six radiomics features, including LeastAxisLength, SurfaceVolumeRatio, Uniformity, InverseVariance, MCC and LargeDependenceLowGrayLevelEmphasis were screened to obtain. Among them, 1 for First Order Features, 2 for Shape Features (3D), 2 for GLCM, and 1 for GLDM. The First Order Features are mainly based on histogram analysis and are used to depict the texture features associated with the gray frequency distribution within the Region of Interest (ROI) (25). In this study, Uniformity belongs to the First Order Features, which describes the image consistency of the ROI. Shape Features (3D) include features describing the size of the ROI and the similarity to a sphere. In this study, LeastAxisLength and SurfaceVolumeRatio belong to Shape Features (3D), which describe the minimum axis length as well as the volume of the ROI. Previous studies have shown that GLCM features are closely related to clinicopathology and can be used to assess the gray-level spatial dependence of ROI as well as to reflect tumor heterogeneity (26). InverseVariance and MCC in this study belong to GLCM features and the texture features derived from them are correlated with the diagnosis of NECs. This is the same as the findings of *Karahaliou* et al. (27) and *Yang* et al. (28) in breast and liver cancers, that GLCM features are sensitive indicators of tumor heterogeneity, and the use of GLCM features can improve the accuracy of diagnosis. GLDM features can also reflect tumor heterogeneity to some extent (29). LargeDependenceLowGrayLevelEmphasis in this study belongs to GLDM features, which can quantify the image grayscale correlation of ROI.

The results of the radiomics nomogram show that the AUC of the ROC curves of the radiomics signature or the radiomics nomogram is higher than the AUC of the ROC curves of the clinical model, and the differences are statistically significant. This implies that contrast-enhanced CT and TNM stage (stage IV) can successfully identify patients with NECs of the digestive system, demonstrating the value of radiomics signature or radiomics nomogram to identify NECs of the digestive system. This can provide a reliable basis for treatment options on the one hand, and a valuable judgment on the prognosis of patients on the other hand. The difference in the AUC of the ROC curves of the radiomics nomogram and the radiomics signature is not statistically significant. This suggests that TNM stage (stage IV) has little role in improving the diagnostic efficacy of NECs of the digestive system and that radiomic signature is more prominent for the diagnostic value of NECs of the digestive system. In addition, the nomogram developed in this study is easy to use and can be used as a tool for individualized preoperative diagnostic prediction of patients.

However, some limitations are inevitable in this study: first, this study was conducted on a malignancy of relatively rare incidence and was a single-center retrospective study with not particularly sufficient cases. Given the great clinical applicability of our findings for the diagnosis of NECs of the digestive system, the next step could be a large-sample multicenter study with more external validation of the constructed model. Second, there was sample selection bias in the retrospective study. Third, clinical characteristics such as age, gender, TNM stage, preoperative CEA and CA199 were included, and the study showed that only TNM stage (stage IV) was associated with the diagnosis for NECs of the digestive system, but the final diagnostic efficacy of the clinical model was still limited, and further exploration with a larger sample of clinical data may be needed in the future. Meanwhile, markers of neuroendocrine differentiation, such as chromogranin A (CgA), neuron-specific enolase (NSE) and synaptophysin (SYP) could be included in the future to allow a more comprehensive analysis of the diagnostic value of clinicopathological features for NECs of the digestive system (30). In addition, this study explored the diagnostic value of contrast-enhanced CT radiomics for NECs of the digestive system, and functional imaging examinations such as somatostatin receptor imaging and 18F-FDG-PET/CT (31) could be included in the future to more systematically assess the diagnostic value of preoperative radiomics for NECs of the digestive system.

In conclusion, we developed a radiomics nomogram that combined radiomics signature and clinical characteristics to effectively diagnose NECs of the digestive system. The nomogram was validated by multiple methods and showed great predictive ability. We expect that the radiomics nomogram can be used as a potential tool to diagnose these patients.

## Data availability statement

The original contributions presented in the study are included in the article/**Supplementary Material**. Further inquiries can be directed to the corresponding authors.

## Author contributions

All authors contributed to data analysis, drafting or revising the article, have agreed on the journal to which the article will be submitted, gave final approval of the version to be published, and agree to be accountable for all aspects of the work. All authors contributed to the article and approved the submitted version.
