# Supplementary material for: Development and validation of a contrast-enhanced CT-based radiomics nomogram for preoperative diagnosis in neuroendocrine carcinoma of digestive system
Source: Front Endocrinol (Lausanne). 2023 Apr 12;14:1155307. doi: 10.3389/fendo.2023.1155307 (PMC10130364; doi:10.3389/fendo.2023.1155307)
Supplement: Supplementary file 1 [file DataSheet_1.docx]

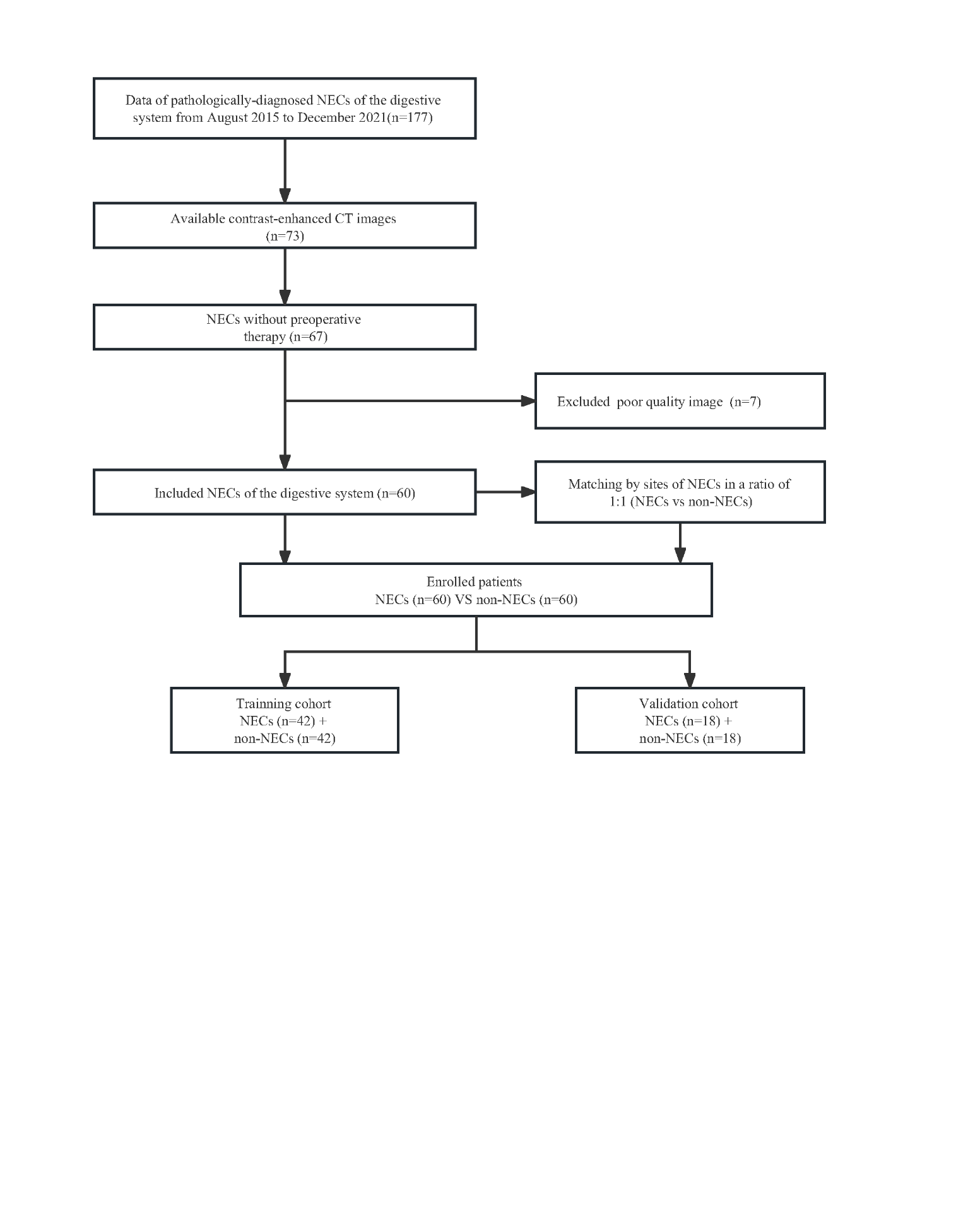


**Supplementary Figure 1. Flow chart for inclusion of patients**

**Supplementary Table 1. Variables and coefficients of the clinical model**

| Variables | β | OR (95% CI) | *p*-value |
| --- | --- | --- | --- |
| Intercept | -0.693 |  |  |
| TNM (II/I) | 0.061 | 0.941 (0.192-5.357) | 0.9416 |
| TNM (III/I) | 0.693 | 2.000 (0.435-11.005) | 0.3873 |
| TNM (IV/I) | 1.917 | 6.800 (1.320-43.164) | 0.0278 |

**Supplementary Table 2. The diagnosis performance of three models in the training and validation cohort.**

| Model | Training cohort | | | Validation cohort | | |
| --- | --- | --- | --- | --- | --- | --- |
|  | AUC (95%CI) | Sensitivity | specificity | AUC (95%CI) | Sensitivity | specificity |
| Clinical model | 0.643 (0.553-0.733) | 0.405 | 0.881 | 0.722 (0.592-0.853) | 0.500 | 0.944 |
| Radiomics signature | 0.893 (0.822-0.965) | 0.833 | 0.833 | 0.867 (0.751-0.983) | 0.889 | 0.778 |
| Nomogram | 0.913 (0.849-0.976) | 0.833 | 0.833 | 0.932 (0.857-1.000) | 1.000 | 0.722 |
